# Locus heterogeneity disease genes encode proteins with high interconnectivity in the human protein interaction network

**DOI:** 10.3389/fgene.2014.00434

**Published:** 2014-12-09

**Authors:** Benjamin P. Keith, David L. Robertson, Kathryn E. Hentges

**Affiliations:** Faculty of Life Sciences, University of ManchesterManchester, UK

**Keywords:** locus heterogeneity, protein interaction network, systems biology, Bardet–Biedl syndrome, Leigh syndrome, Kabuki syndrome

## Abstract

Mutations in genes potentially lead to a number of genetic diseases with differing severity. These disease genes have been the focus of research in recent years showing that the disease gene population as a whole is not homogeneous, and can be categorized according to their interactions. Locus heterogeneity describes a single disorder caused by mutations in different genes each acting individually to cause the same disease. Using datasets of experimentally derived human disease genes and protein interactions, we created a protein interaction network to investigate the relationships between the products of genes associated with a disease displaying locus heterogeneity, and use network parameters to suggest properties that distinguish these disease genes from the overall disease gene population. Through the manual curation of known causative genes of 100 diseases displaying locus heterogeneity and 397 single-gene Mendelian disorders, we use network parameters to show that our locus heterogeneity network displays distinct properties from the global disease network and a Mendelian network. Using the global human proteome, through random simulation of the network we show that heterogeneous genes display significant interconnectivity. Further topological analysis of this network revealed clustering of locus heterogeneity genes that cause identical disorders, indicating that these disease genes are involved in similar biological processes. We then use this information to suggest additional genes that may contribute to diseases with locus heterogeneity.

## INTRODUCTION

The characterization of mutations in genes that cause human genetic disease is vitally important. Once identified, these mutant genes (termed disease genes) provide an opportunity to study the origins of genetic disorders and develop potential therapeutics to mitigate symptoms or deliver curative strategies targeting these genes. In recent years, the discovery and classification of disease genes within the human genome has received increasing attention. As databases of disease gene associations, such as the Online Mendelian Inheritance in Man (OMIM; Hamosh et al., 2005), continue to increase in size and accuracy, we can use these data to further understand disease pathogenesis. In a previous study (Dickerson et al., 2011) we found that disease genes do not form a homogeneous group of genes with shared characteristics – but instead cluster into distinct groups each with shared characteristics. Isolating genes displaying similar attributes may therefore lead to the discovery of further associated gene groups, allowing us to examine their relationship with disease.

**FIGURE 1:**
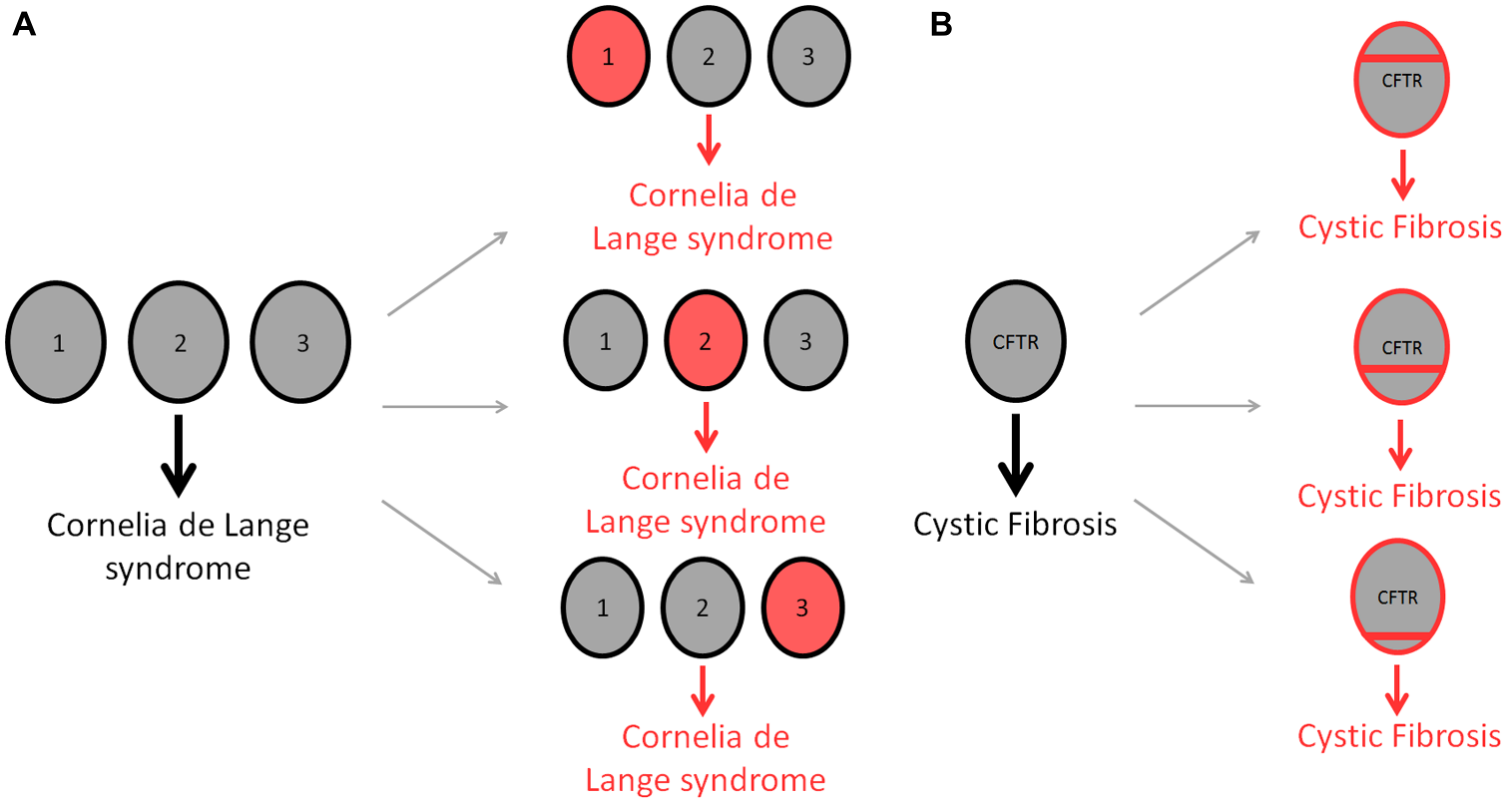
**Differences in genetic heterogeneity.** Locus heterogeneity describes the ability of identical disorders to be acquired through mutations in a number of different genes **(A)**. Gray circles represent wild-type genes, whereas red circles denote mutated genes. The developmental disease, Cornelia de Lange syndrome, can be acquired through a single mutation in any of three different genes; NIPBL (1), SMC1A (2) or SMC3 (3), producing the same disorder in each case (Liu and Baynam, 2010). Allelic heterogeneity describes the ability of different mutations within the same gene to cause the same disease **(B)**. Cystic fibrosis is used to demonstrate this form of heterogeneity, with as many as 1,500 CFTR mutations being attributed to causing the disorder (O’Sullivan and Freedman, 2009). Red bars indicate different mutations within the CFTR gene.

Is it now appreciated that human disease is characterized by genetic heterogeneity, for which two different types exist. Allelic heterogeneity refers to instances where mutations in different alleles at the same locus produce the same disease. By contrast, locus heterogeneity describes mutations in different genes whereby any one mutation generates the same disorder (**Figure 1**; McClellan and King, 2010). Many genetic diseases display locus heterogeneity, with affected genes being associated with almost all disease categories and cell types. Perhaps the most striking example of locus heterogeneity is the disorder retinitis pigmentosa, a retinal dystrophy resulting from the loss of photoreceptors in the retina for which more than 45 genes have been identified (Hartong et al., 2006). A number of recent studies into the mechanisms by which these genes cause identical disorders suggest that protein products of affected genes are likely to be functionally similar, interacting with one another and displaying an involvement in the same biological pathways and processes (Wang et al., 2012; Guo et al., 2013). With this is mind, an appropriate method to study the associations between genes involved in these disorders is to investigate the complex interconnections between cellular components.

The advent of high-throughput, ‘omic’ technologies in the last decade has resulted in rapid growth in the number of identified and mapped protein interactions available within interaction databases. For example, BioGRID (Stark et al., 2006) provides genetic and biological interaction data for a range of species and the Human Protein Reference Database (Peri et al., 2004) curates literature sourced human protein interactions. Although by no means complete, these individual “building blocks” have been used to construct biological networks, ranging from small cellular systems to genome-wide interactomes. Through examining the topological properties of these networks, we can gain insights into the complex relationships between proteins, and therefore disease-associated proteins, in a branch of computational biology commonly referred to as “network medicine” (Barabasi et al., 2011; Thanh-Phuong and Tu-Bao, 2012).

Existing network analysis based studies have utilized the analytical advantages of interaction networks to reveal the highly interconnected relationships between genes expressing locus heterogeneity. A study by Bauer-Mehren et al. (2011) used an extensive gene-disease association database to create a gene-disease network to examine how pathway perturbations result in disease phenotypes, with the aim of assessing whether modularity applies to a spectrum of different disorders. Modularity was observed for genes of all disorder types, including those that expressed locus heterogeneity. A more specific study considered the “pathogenic” genes of functional pathways in autism spectrum disorder (ASD) and intellectual disability (ID), an array of disorders caused by heterogeneous gene mutations (Krumm et al., 2014). This study showed, as previously hypothesized, that locus heterogeneity genes associate within close proximity to one another in biological pathways, and contribute highly similar functional roles to their respective systems.

In this study we tested the hypothesis that within protein interaction networks, locus heterogeneity genes are more highly interconnected to other genes causing the same disorder than genes associated with Mendelian diseases or non-disease genes. Throughout, locus heterogeneity disorders were classed as those caused by mutations in a number of genes, but inherited in a monogenic/simple fashion. Complex heterogeneous disorders caused by mutations in multiple alleles acting together were not considered here. To complete our investigations we manually curated a number of locus heterogeneous disorders and their associated genes. We generated a global human protein interaction network from various human interaction databases. By considering the local neighborhood of heterogeneous genes, we were able to identify potential novel locus heterogeneity genes involved in specific disorders. A comparison of the locus heterogeneity curated genes with those that cause single-gene Mendelian disorders served as a method to isolate and identify properties of locus heterogeneity genes. The results of this study demonstrate that locus heterogeneity genes display distinct network properties, forming clusters of disorder specific genes. These network clusters can be utilized to suggest novel disease genes for further experimental studies.

## MATERIALS AND METHODS

### DATA RETRIEVAL

Disease genes were parsed from the OMIM database genemap (03/02/2014 update; Hamosh et al., 2005) and filtered according to ‘confirmed’ genes (observed in at least two laboratories). Disease genes that had no disease annotation in the “disorder” field of the genemap were also filtered. A dataset of 5671 disease genes was produced from this process, of which 2485 could be mapped onto the protein interaction network.

Disease gene data relating to heterogeneous and Mendelian disorders were obtained from a combination of ResNet (10/02/2014 update; Daiger et al., 1998), a database providing genetic data relating to a number of retinal disorders, and Genetics Home Reference (GHR; Fomous et al., 2006), a resource of integrated clinical information that curates disorder specific research to provide information for patients. The selection of both heterogeneous and Mendelian disorder was aided by a number of review articles (McKusick, 1991; Chial, 2008; McClellan and King, 2010) that classify the properties of genetic disorders, and using this information, along with GHR to find related verified disorders sharing the same inherited properties. Final datasets for heterogeneous and Mendelian disorders contained 674 and 397 genes respectively.

Human protein–protein interaction data was retrieved using ConsensusPathDB (CPDB, release 28; Kamburov et al., 2013), an integration of 32 public interaction resources to provide a high quality consensus of available protein interaction data. The full dataset, containing 16363 nodes and 179685 edges, was used for comparison and analyses throughout. Conversion of protein IDs from official gene symbol to UniProt ID was performed with the gene ID conversion tool of DAVID Bioinformatics Resources (version 6.7; Huang et al., 2009a,b) prior to the mapping of disease genes onto the ConsensusPathDB (CPDB) network.

### DISEASE CATEGORIZATION

Genes were classified into appropriate disease categories using the Medical Subject Headings controlled vocabulary (MeSH; Lowe and Barnett, 1994). High level terms were merged with classifications used in Goh et al. (2007) and Dickerson and Robertson (2012) to present 20 unique classifications representing a wide range of physiological systems.

### NETWORK VISUALIZATION AND TOPOLOGICAL ANALYSIS

Protein–protein interaction networks were visualized and analyzed using Cytoscape (version 2.8.3 and version 3.1.0; Shannon et al., 2003). All networks presented here are undirected and use the edge-weighted spring embedded layout, unless otherwise stated, and have had self-loops and duplicated edges removed. The Cytoscape plugin AllegroLayout (AllegroViva Inc, 2014a) was used to produce spring-embedded visualization of the network. NetworkAnalyzer was used to verify network properties such as degree (total number of edges connecting to one node), degree distribution (the probability distribution of all degrees within the network) and clustering coefficient (the measure to which nodes within the network tend to cluster together; Barabasi and Oltvai, 2004) within Cytoscape 3.1.0.

Topological analysis of the network was achieved within Cytoscape using the clustering tool AllegroMCODE 2.1 (AllegroViva Inc, 2014b). Clusters with an MCODE (Bader and Hogue, 2003) complex score higher than 3 were chosen for further study. Default settings were used, unless otherwise stated.

Additional methods were utilized to validate selected clusters. The Louvain method for network community analysis attempt to reveal a hierarchical structure for larger networks, discussed in Blondel et al. (2008).The overlapping clustering algorithm, EAGLE (Shen et al., 2009), and the clustering coefficient-based clustering algorithm, FAG-EC (Li et al., 2009), were also applied. The Louvain method was implemented using a command line tool (Blondel et al., 2008), while EAGLE and FAG-EC were applied using the Cytoscape plugin ClusterViz (Wang et al., 2014). Default settings were used throughout our analyses.

### GENE FUNCTIONAL AND PATHWAY ANALYSIS

Identifying key properties of unannotated genes found with disease enriched clusters was achieved using Ingenuity Pathway Analysis (IPA; Ingenuity® Systems, 2014). IPA was also utilized to identify over represented signaling or metabolic canonical pathways to propose further similarities between genes and proteins within network clusters.

The Cytoscape plugin BiNGO 3.0.2 (Maere et al., 2005) was used to retrieve Gene Ontology (GO) annotations (Ashburner et al., 2000), mapping them onto data within Cytoscape directly. As in Dickerson et al. (2011), GO entries with their molecular function category marked with the term “activity” were used for functional analysis of the global network as well as individual clusters.

### STATISTICAL ANALYSIS

Statistical analyses were performed using R-Development-Core-Team (2009). Pearson’s Chi-squared test was used to assess whether disease classifications was significantly different between heterogeneity and Mendelian datasets. The Benjamini and Hochberg False Discovery Rate was used to calculate corrected p-values for GO functional classification testing to minimize multiple comparison errors.

A Perl script utilizing the Graph module (Hietaniemi, 2014) was written to determine the significance of locus heterogeneity gene related observations within our network. This script calculated the proportion of locus heterogeneity nodes having a locus heterogeneity neighbor, and determined significance through 10,000 randomizations of the dataset, assigning heterogeneity to the same number of nodes and assessing the proportion for this randomized dataset compared to the real observed dataset. For each randomization we assumed that the average topology of heterogeneous nodes is the same.

## RESULTS

### LOCUS HETEROGENEITY AND MENDELIAN DISORDER CLASSIFICATION

**FIGURE 2:**
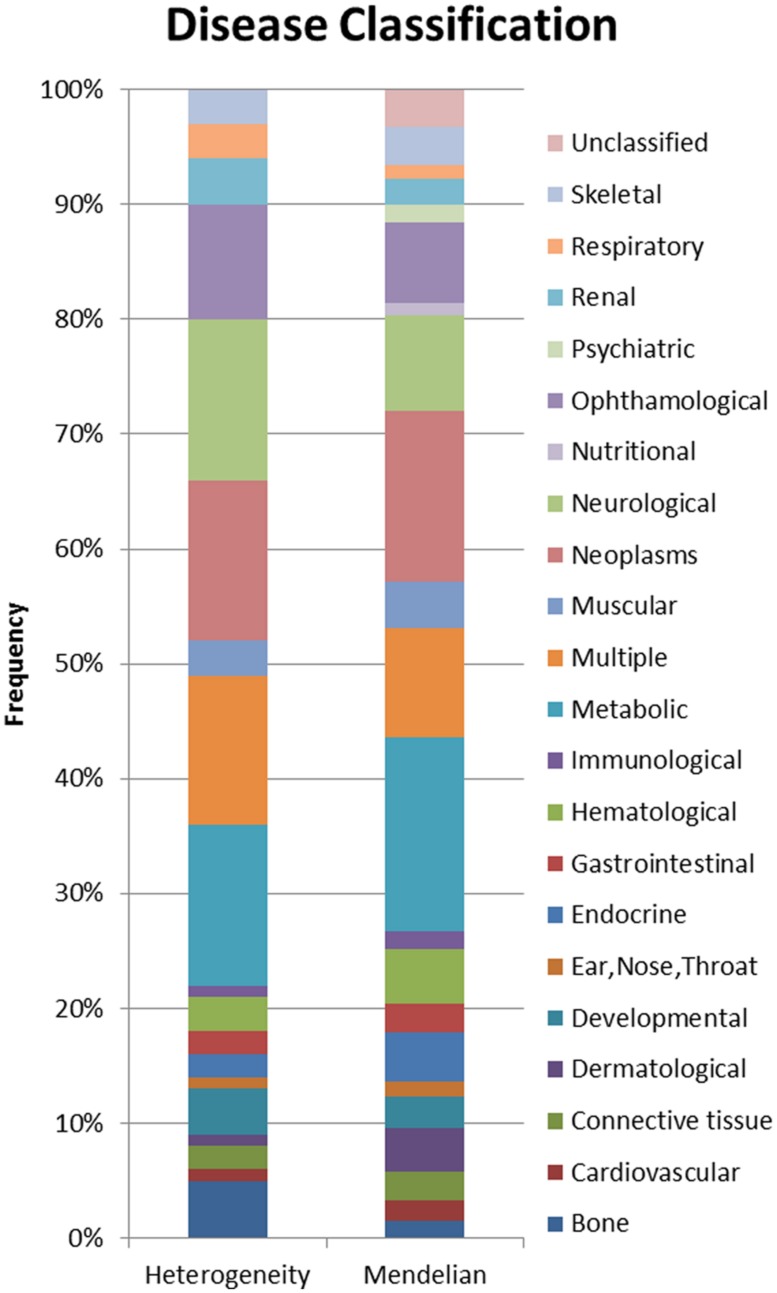
**Proportional display of diseases by MESH classification.** The proportion of locus heterogeneity (left) and Mendelian (right) disease genes characterized in our study that affect different physiological systems. Colors correspond to specific physiological systems affected by these disease genes (key at far right).

Using a combination of OMIM’s genemap (Hamosh et al., 2005) and disease specific databases (Daiger et al., 1998; Fomous et al., 2006), disease genes (*n* = 2485), locus heterogeneity genes (*n* = 674), and Mendelian genes (*n* = 397) were selected based on etiological information accompanying human disorders. MeSH classifications were applied to locus heterogeneity and Mendelian genes to identify disease types associated with the two datasets. This allowed us to examine differences in the physiological systems affected by the diseases (**Figure 2**), which might impact upon our analysis.

In order to prevent any potential bias, we chose Mendelian disease genes to include in our dataset because they shared the same disease classification proportions as our locus heterogeneity genes. It was not possible to eliminate all variation between the two datasets, however, these differences have been minimized by the selection of Mendelian disorders affecting the same physiological systems as those affected in diseases showing locus heterogeneity. A Pearson’s Chi-squared test confirmed that the two datasets were not significantly different in the systems affected (*p* = 0.372).

### LOCUS HETEROGENEITY NETWORKS SHOW DISTINCT PROPERTIES COMPARED TO OTHER DISEASE-ASSOCIATED NETWORKS

**FIGURE 3:**
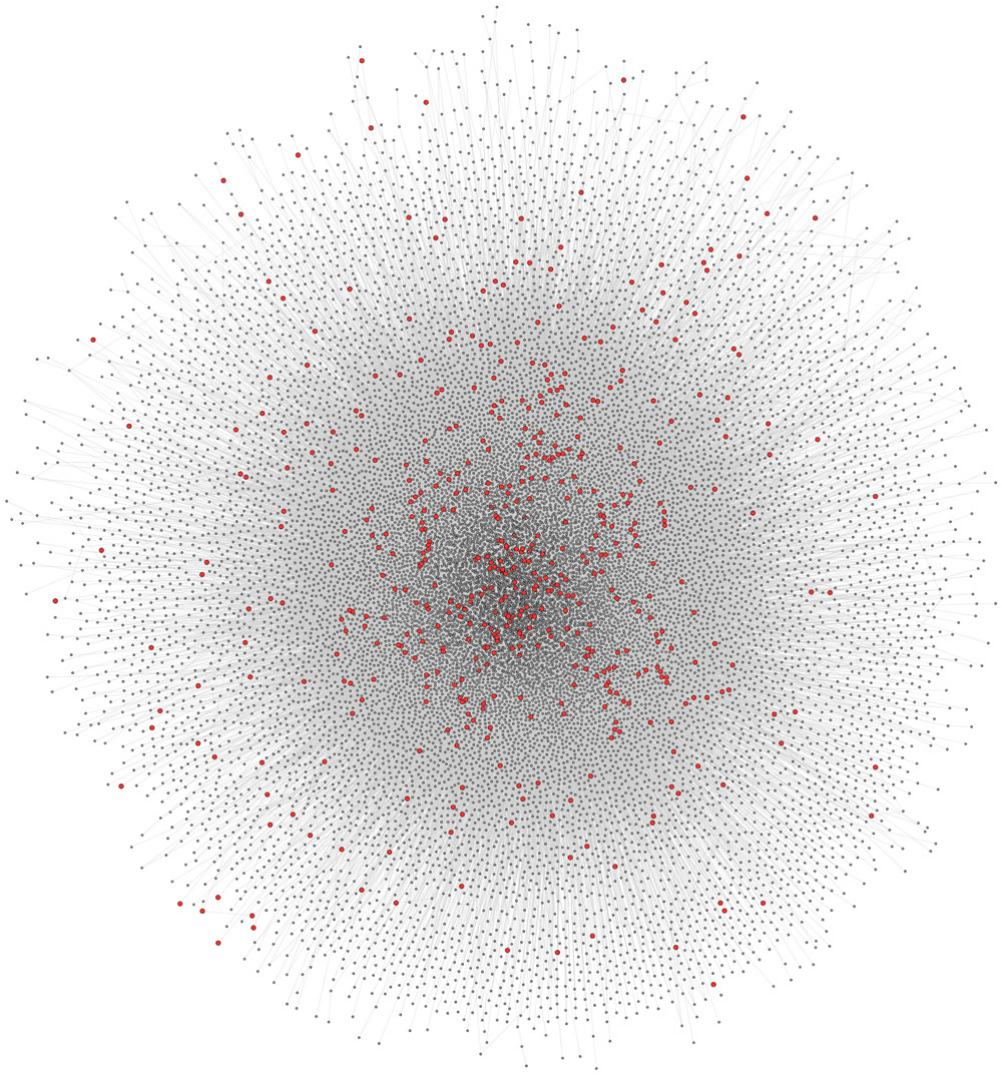
**Full CPDB protein interaction network.** The network displays the full set of interactions available from CPDB used in this study. Circles (nodes) represent proteins, whereas the lines (edges) connecting two circles signify an interaction between two proteins. Locus heterogeneity genes relating to our 100 selected disorders are highlighted red, with gray nodes symbolizing other genes in the dataset.

The full human protein–protein interaction network was retrieved from CPDB, consisting of 16363 nodes and 179685 edges (**Figure 3**). Since this interaction data is sourced from a number of interaction databases and experimental studies, the resulting collection of data contains protein interactions from multiple sources, such as co-immunoprecipitation and yeast two-hybrid studies. To extract and analyze specific networks in isolation, the proteins encoded by disease genes, locus heterogeneity genes and Mendelian genes were mapped onto the network. Although a total of 674 locus heterogeneity genes and 397 Mendelian genes were identified from ResNet (Daiger et al., 1998) and Genetic Home Reference (Fomous et al., 2006), 13 locus heterogeneity and 32 Mendelian disease genes could not be translated onto the interaction network. Redundancy among disease genes was the cause of the majority of genes losses after mapping, as exemplified through the diseasome bipartite network in Goh et al. (2007). For example, the single disease gene *ERCC2* causes both trichothiodystrophy and xeroderma pigmentosum. Other potential causes for this decrease in gene numbers include errors in gene ID conversion between gene naming conventions and unavailable protein interaction data, either due to missing data within the database or a current lack of experimental interaction data. We found differences between the three categories of disease genes, confirming that heterogeneous genes display network topology properties different to that of the disease gene population as a whole, and to those of Mendelian disease genes (**Table 1**).

**Table 1:** Disease network parameters.

	Full networks			Largest connected component		
	Full disease	Heterogeneity	Mendelian	Full disease	Heterogeneity	Mendelian
Number of nodes	2485	535	301	2040 *(82.1%)*	323 *(60.4%)*	134 *(44.5%)*
Average degree	7.305	2.931	1.362	8.881	4.669	2.866
Isolated nodes	415 *(16.7%)*	163 *(30.5%)*	148 *(49.2%)*	N/A	N/A	N/A
Network centralization	0.113	0.128	0.049	0.137	0.207	0.100
Clustering coefficient	0.119	0.141	0.049	0.145	0.233	0.094

*Network properties were calculated for each of the three full disease networks, and the largest connected component of these networks. The LCC calculations ignore isolated nodes and clusters. For specific parameters, the percentage of nodes within the full network displaying each property is listed in parentheses.*

Analysis was performed on both the full network and the largest connected component (the largest interconnected group of nodes within the network, LCC) to exclude disconnected nodes. Initial parameter calculations revealed a large percentage of isolated nodes (nodes with a degree value of 0) within the three networks. As detailed in previous studies (Hirschhorn and Daly, 2005; Bauer-Mehren et al., 2011), human inherited diseases arise due to genetic mutations that disrupt the complex interactions between network components. Although parameter calculation using the LCC may provide a more accurate representation of disease gene connectivity, perhaps correcting for any bias introduced as a result of unavailable interaction data, a high number of isolated nodes within these specific networks provides vital information. The smaller percentage of Mendelian disease genes within the largest connected component (44.5%) in comparison to the full disease (82.1%) and locus heterogeneity (60.4%) networks, suggests that Mendelian disease genes are not as interconnected as other disease genes.

In both the full networks and the LCC networks, average degree (the average number of interactions across all nodes) is largest in the disease network and lowest in the Mendelian network. Although the full disease network has a larger average degree, we would expect to observe clustering in the heterogeneous network due to the perturbation of different genes causing identical disorders as a result of their functional pathway similarities (Guo et al., 2013). Network centralization is a relative measure of node isolation, and describes how nodes are connected on the scale of the whole network (Dong and Horvath, 2007). The locus heterogeneity network has a larger centralization measure than the total disease network and the Mendelian network for the full networks, and the LCC (**Table 1**). This larger centralization score implies that the heterogeneous network is more densely connected compared to the other networks.

Additionally, we analyzed clustering in the various disease networks. The average clustering coefficient characterizes the tendency of nodes to form highly connected clusters, used previously by Ravasz et al. (2002) to study the modular organization of metabolic networks. Our data show that the locus heterogeneity network has the largest average clustering coefficient of the three disease networks for both the full network and the LCC. This suggests that locus heterogeneity genes form groups of highly interconnected clusters, confirming the prediction that gene-products causing the same disorder interact with each other.

### LOCUS HETEROGENEITY GENES SHOW SIGNIFICANT INTERCONNECTIVITY WITHIN THE GLOBAL PROTEIN INTERACTION NETWORK

To investigate the connectivity of locus heterogeneity associated proteins within the full CPDB interaction network, we utilized the Perl module package Graph (Hietaniemi, 2014) to allow the calculation of gene connectivity, and to perform randomizations by assigning heterogeneity to the same number of a random set of proteins and testing the resulting connectivity. We performed 10,000 random simulations and calculated the percentage of locus heterogeneity proteins connected to another locus heterogeneity protein with the network for comparison to the true dataset.

**FIGURE 4:**
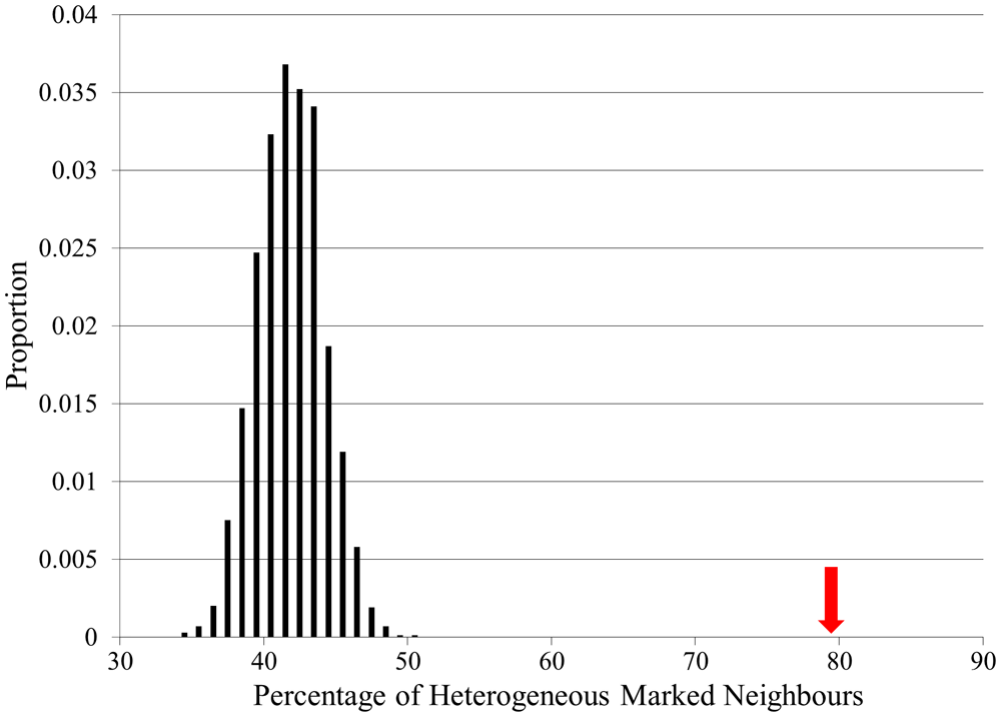
**Locus heterogeneity gene interconnectivity within the full CPDB network compared to random simulations.** There is a normal distribution of random simulations (black bars), with a mean value of 41.9%. The red arrow indicates the actual percentage connectivity of locus heterogeneity genes (79.7%), showing a significant difference from 10,000 random simulations.

The connectivity of actual locus heterogeneity proteins within the network was 79.7%, which was significantly higher than the connectivity in any of our random simulations, which displayed a mean connectivity value of 41.9% (*p* < 0.0001; **Figure 4**). Whilst showing that the connectivity of locus heterogeneity genes is higher than expected by chance, this test also further confirms the high degree of connectivity of heterogeneity genes within our interaction network.

Using this same method to examine the connectivity of proteins associated with single-gene Mendelian disorders produced a significant result, although in this case the initial connectivity percentage was 64%. This interconnectivity between Mendelian genes may be due to the large number of Mendelian disease genes in our dataset affecting the same physiological systems (**Figure 2**). However, proteins associated with locus heterogeneity are more connected than proteins associated with Mendelian disease (79.7% compared with 64%), despite both sets of disorders showing an equal distribution of physiological pathologies. This further emphasizes the greater interconnectivity of disease-associated locus heterogeneity genes compared to disease-associated Mendelian genes.

### CLUSTERING ANALYSIS OF THE HUMAN PROTEOME REVEALS HIGHLY INTERCONNECTED MODULES OF LOCUS HETEROGENEITY GENES

Clustering analysis was performed on protein interaction networks in an attempt to find protein complexes and functional clusters, which can be identified as highly interconnected subgraphs. Topological modules signify areas of dense local connectivity within a network, and with the use of experimental data, can be validated as functional modules of proteins defining an aggregation of proteins with similar or related biological function (Vidal et al., 2011). Here, a pre-existing algorithmic approach was used to identify densely interconnected groups within the locus heterogeneity disease network, and through the application of IPA and GO, we were able to confirm the functional relatedness of these genes.

As suggested by the average clustering coefficient of the locus heterogeneity disease network, we found that locus heterogeneity genes responsible for the same disease tended to be highly interconnected, and were present in the same topological modules. This result provides additional evidence for the highly interconnected nature of locus heterogeneity proteins. We further predict that a number of proteins within these modules positioned in close proximity to a group of locus heterogeneity proteins may be involved in the pathology of similar disorders, or may in fact be an undiscovered cause for locus heterogeneity disorders. The following examples [Bardet–Biedl syndrome, Leigh syndrome (LS), and Kabuki syndrome (KS)] demonstrate how genes within the local modular neighborhood of a locus heterogeneity disease gene may be possible disease gene candidates.

These functional modules displayed an MCODE complex score higher than 3, which indicates a greater accuracy and reliability of predictions. To determine if the clustering algorithm altered the modules produced from the network, modules were validated using alternative clustering algorithms. The Louvain method (Blondel et al., 2008), EAGLE (Shen et al., 2009), and FAG-EC (Li et al., 2009) provided alternative implementations of clustering within our network, but still produced our three locus heterogeneity disease modules. Further examples of modules identified, but not covered here, can be found in the supplementary data.

#### Bardet–Biedl syndrome

Bardet–Biedl syndrome (BBS) is a genetically and clinically heterogeneous disorder of developmental origin caused by mutations in a number of loci, with primary features including retinal dystrophy, hypogenitalism, renal malformations, and obesity (Badano et al., 2003). A number of studies have highlighted that the primary cause of BBS is ciliary dysfunction, and it is noted as one of the first disease to have an etiology associated with epithelia dysfunction (Zaghloul and Katsanis, 2009). Genes involved in this disorder are therefore suspected to play vital roles in cilia structures within cells (Baker and Beales, 2009). For example, genes associated with BBS are vital for sensory perception (such as hearing and sight), with BBS gene products displaying an involvement in the maintenance and function of cilia (Baker and Beales, 2009). Mutations in BBS genes lead to defects in cell structures important in chemical signaling pathways, causing aberrations of regular sensory perception (Tobin and Beales, 2007).

**FIGURE 5:**
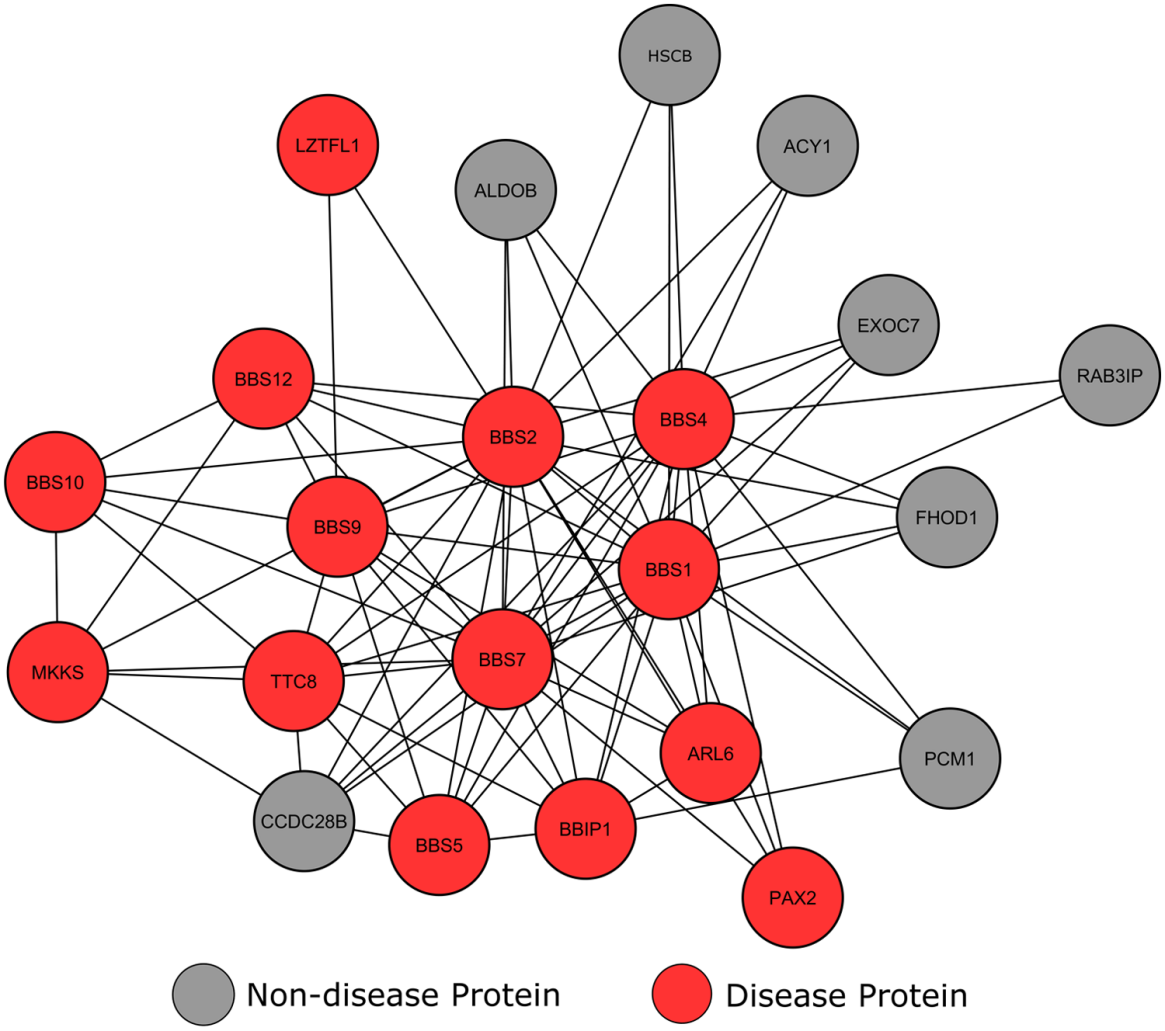
**Interconnectivity of Bardet–Biedl syndrome genes.** Circular nodes represent proteins, with the lines between them signifying an interaction between the two proteins.

Clustering analysis of our network using the MCODE algorithm (Bader and Hogue, 2003) revealed a high scoring cluster, in which many nodes were tagged as BBS affected proteins (**Figure 5**). This module shows a number of locus heterogeneity genes (red), all of which encode BBS causing proteins. Surrounding nodes for genes not currently associated with BBS (gray) interconnect with a minimum of two BBS causing genes, suggesting a potential involvement in or cause of BBS for these other connected genes.

The most highly connected of these ‘healthy’ proteins is CCDC28B. Literature searches confirmed that this gene-product has known involvement in an alternative form of BBS. BBS is usually inherited in a monogenic autosomal recessive manner; in rare cases three mutations across two loci modify the onset and severity of the phenotype. Along with genes already annotated within our dataset, studies have shown that CCDC28B is one of these modifier genes (Beales et al., 2003; Badano et al., 2006).

The proteins in our network currently lacking in BBS annotations preferentially connect with BBS1, BBS 2, BBS4 and BBS7, with the exception of PCM1, which also interacts with BBIP1. According to GO analysis, a number of these proteins are involved in cilium assembly (*p* = 7.37e-18) and epithelial neoplasia (*p* = 1.21e-22), similar to known BBS causing genes. The molecular chaperone HscB only has three characterized protein interactions, all of which are with BBS causing proteins. The HscB protein displays similar cellular localization and interactions with BBS proteins, and previous studies have shown the HscB mutations have the ability to cause protein folding malformations (Vickery and Cupp-Vickery, 2007). Therefore, these data suggest that HscB may be a potential BBS candidate. Another protein with no current disease annotations is RAB3IP. A number of studies have shown that core BBS proteins form a complex that cooperate with GTPases, including RAB3IP, to promote ciliary membrane biogenesis (Nachury et al., 2007; Westlake et al., 2011). Nachury et al. (2007) used zebrafish to show that blocking of GTPase production prevents ciliogenesis in cells, yielding BBS-like phenotypes. Although this is yet to be proven in humans, the interconnectivity between RAB3IP and known BBS causing genes suggests that RAB3IP may be a candidate BBS causing gene.

#### Leigh syndrome

Leigh syndrome is characterized by severe neurodegeneration arising typically within the first year of life, manifesting clinically through rapid deterioration of cognitive and motor functions due to lesions in the basal ganglia and brain stem of affected patients, with clinical and genetic heterogeneity (Finsterer, 2008; Baertling et al., 2014). Since LS is classed strictly as a mitochondrial disorder, associated mutations affect genes connected with the mitochondrial and nuclear genomes, making the discovery of genes suspected to be involved in the disorder challenging (Finsterer, 2008). In healthy individuals, wild-type forms of LS genes are involved in energy production in the mitochondria. Many gene mutations associated with LS disrupt protein complexes that are vital to the process of oxidative phosphorylation, therefore preventing maximal energy production by the mitochondria. Other mutations known to cause LS also act to obstruct protein complexes involved inoxidative phosphorylation, or other processes relating to energy production (Gerralds, 2014).

**FIGURE 6:**
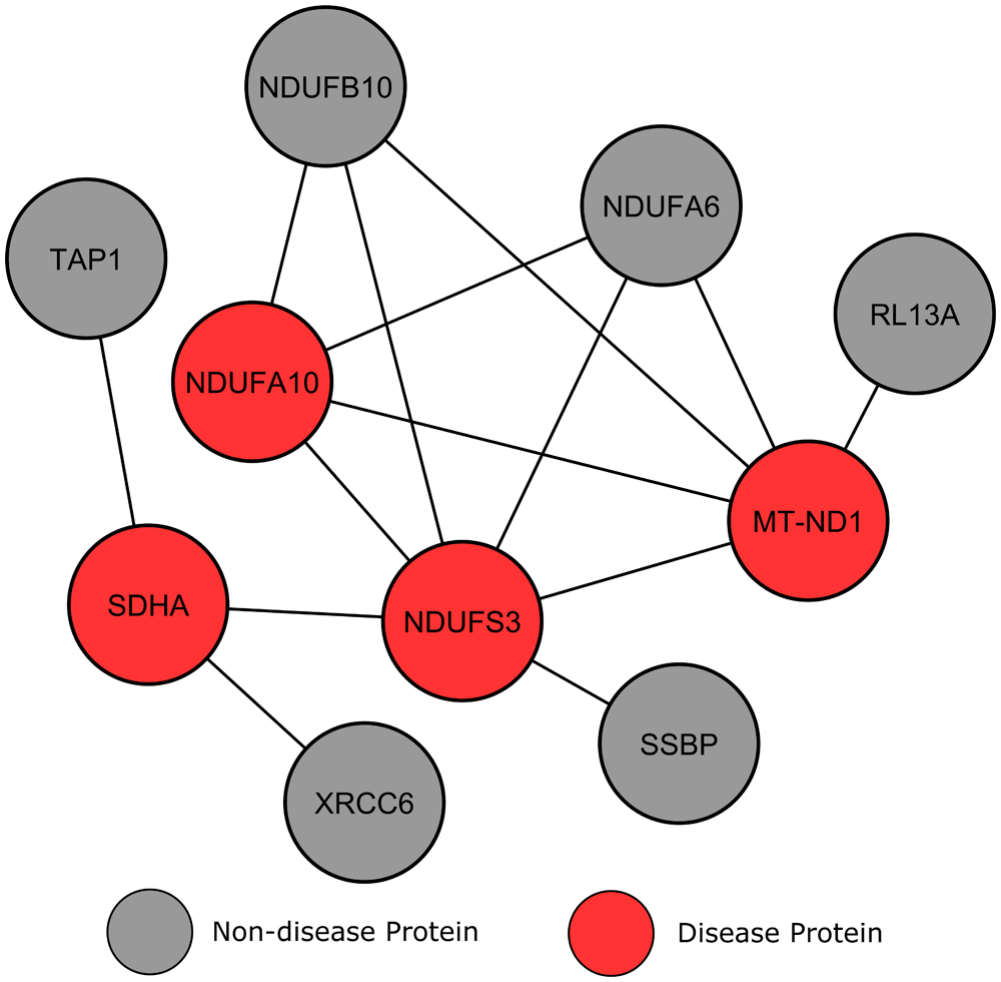
**Leigh syndrome gene clustering.** Each circular node denotes a protein and a line illustrates an interaction between two proteins.

The module shown in **Figure 6** involves four LS affected proteins surrounded by a number of proteins without disease annotations. Compared to the previous example, these non-disease proteins show a more varied connection to locus heterogeneity proteins. Two proteins, NDUFA6 and NDUFB10, both connect to three LS genes and, according to IPA, belong to the identical canonical pathways as these three LS affected proteins (mitochondrial dysfunction and oxidative phosphorylation). Further inspection using GO analysis confirmed that the two unmarked proteins are involved in the same biological processes as our LS causing genes, for example the respiratory electron transport chain (*p* = 6.94e-15). Previous studies analyzing these mitochondrial enzymes have suggested that they have an involvement in neurodegeneration, and that their perturbation may play a role in neurodegenerative disorders (Harris et al., 2007; Kaltenbach et al., 2007; Satoh et al., 2013). Therefore, the interconnectivity of NDUFA6 and NDUFB10 proteins with LS causative proteins implies that specific mutations in these genes may produce an LS phenotype.

In contrast, other surrounding genes only connect to one LS protein and show less connectivity to the disorder, therefore making them less likely to be disease candidates. Although these proteins localize to the mitochondria, they are not found in the same canonical pathways (mitochondrial dysfunction and oxidative phosphorylation) as known LS genes. This result suggests that genes with a higher degree of connectivity to multiple heterogeneous genes increases the likelihood of that gene’s involvement in the same biological processes, and therefore increases a gene’s potential to be a disease candidate.

#### Kabuki syndrome

Whilst the two disorders discussed previously show severe locus heterogeneity, KS is only known to occur through mutations in the histone methyltransferases KMT2D (also known as MLL2) or KDM6A, causing a breakdown in the epigenetic control of active chromatin states (Hannibal et al., 2011). KS is a congenital disorder presenting with multiple malformations of the facial area, ID and cardiac defects (Bokinni, 2012). In a number of cases, KS patients have no identified KMT2D or KDM6A gene mutations (Miyake et al., 2013). Whilst in these cases the cause of the disorder is unknown, these additional cases indicate that the disorder may show further heterogeneity (Bokinni, 2012). Wild-type KS genes produce enzymes that function as histone methyltransferases, regulating the activity of genes in many of the body’s organs and tissues. The absence of these functional enzymes therefore prevents the correct activation of several genes, leading the physiological abnormalities observed in KS patients (Bokinni, 2012).

**FIGURE 7:**
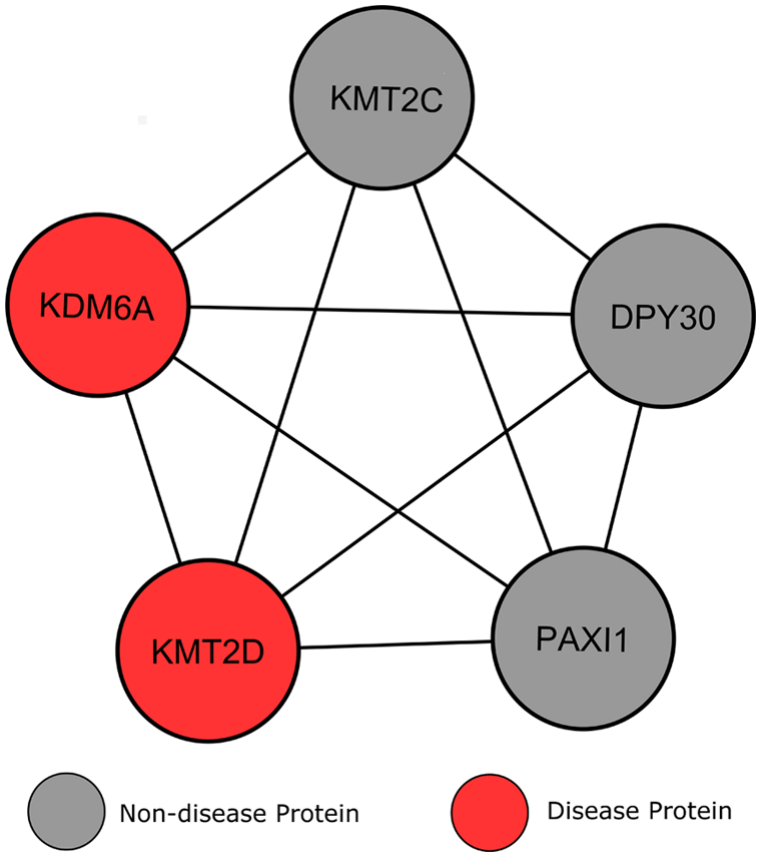
**Heterogeneity of genes causing Kabuki syndrome.** Lines signify interactions between two proteins, represented by circular nodes.

Clustering analysis revealed a module whereby five proteins interconnect with one another, including the two KS associated proteins (**Figure 7**). Additionally, according to GO analysis all proteins within this submodule localize within the nucleus, specifically within histone methyltransferase complexes (*p* = 3.56e-7), and have identical biological processes in chromatin modification (*p* = 2.19e-7). Perhaps the most interesting of these connected genes is KMT2C (MLL3), a lysine-specific methyltransferase that acts in a similar manner to KMT2D, and has recently shown strong associations to other neoplasmic disorders (Li et al., 2013a,b). DPY30 is another of these connected proteins, for which experimental evidence is limited in humans. Jiang et al. (2011) suggests a role for DPY30 in histone methyltransferase complex regulation, but its function in human disease is yet to be fully explored. The final of these connected proteins, PAXI1, associates with methyltransferases to maintain genome integrity during gene rearrangements and has been labeled as “the gatekeeper of thymocyte development” (Callen et al., 2012; Papatriantafyllou, 2013). Callen et al. (2012) have shown that PAXI1 has specific roles in DNA repair and transcription to prevent oncogenic DNA damage.

Current knowledge of the roles of the genes KMT2C, KMT2D, and PAXI1, along with their interconnectivity with the two known KS proteins and evidence in the literature that KS may be caused by mutations in additional genes, suggests that these genes should be the target of genetic screening in patients where KMT2D and KDM6A mutations have not been detected.

## DISCUSSION

Our study demonstrates that disease genes expressing locus heterogeneity display properties that allow them to be distinguished from disease genes causing simple Mendelian disorders, such as sickle cell anemia, and disease genes as a whole. Analysis of the human proteome revealed that proteins encoded by locus heterogeneity genes are highly interconnected with those involved in the same disorder, grouping together in the clustering analysis of the network (**Figures 5**–**7**). In agreement with a study by Bauer-Mehren et al. (2011), we found that locus heterogeneity genes display modularity and tend to associate within the same biological pathways, suggesting that these disorders are associated with a set of biological pathways, rather than single pathways. As suggested by Furlong (2013) the modularity observed by locus heterogeneity genes is similar to those involved in a numbers of cancers, including breast (Walsh and King, 2007) and pancreatic cancers (Jones et al., 2008), whereby the same cancer type can be the result of mutations in a number of different genes. Walsh and King (2007) have suggested, because these genes converge on specific biological functions, that there are still other breast cancer genes to be identified. Examining the clustering and connectivity of genes connected to known cancer genes within biological networks provides an opportunity to reveal candidate disease genes to promote further study and investigation.

The techniques employed here have been used in recent studies concerning ASD and ID, a group of disorders that display considerable locus heterogeneity (O’Roak et al., 2012; Krumm et al., 2014). Protein interaction networks have been used in these studies to show the significant enrichment of *de novo* mutations in a group of Fragile-X syndrome genes (Iossifov et al., 2012), and to demonstrate that previously identified ASD and ID risk genes have a reduced network distance, therefore being more closely associated in the network (Neale et al., 2012). Most notably, O’Roak et al. (2012) mapped ASD genes from patient exome data onto a protein interaction network to show that the most severe *de novo* mutations mapped to a highly interconnected network significantly enriched for autism candidate genes. As well as further confirming the “extreme” locus heterogeneity of ASD, these results have provided a pathway for future discovery.

The use of protein interaction networks in this study allowed for large-scale comparisons of 1000s of protein interactions curated from a number of experimental sources. Despite the ability to easily identify relationships between genes, and the extent to which proteins interconnect, these networks, and the methods used to analyze them, have important limitations which must be considered. Firstly, even though interactions within the network have been experimentally verified from a number of sources, protein interactions are often difficult to assay on a proteomic scale, leading to false negative and false positive results. As well as an inability to distinguish between transient and obligate interactions within the network, data concerning the spatial and temporal nature of interactions is often limited or ignored for network reconstructions such as this. Finally, the importance of particular interactions can vary between nodes, even within clusters, meaning that experimental validation of candidate predictions is vital (O’Roak et al., 2012). Integrating various layers of experimental data will become common practice in future studies, and will facilitate the production of networks that are more biologically representative of the systems they are modeling. A potential difficulty when using clustering algorithms on networks of this scale is the reliability of the results obtained. This can be alleviated through using sensible score thresholds, along with multiple clustering methods to remove any spurious results (Barabasi et al., 2011). In this study, we used a score threshold determined through comparisons of theoretical and experimentally derived protein pathways and complexes (Bader and Hogue, 2003). We then implemented three alternative clustering methods to increase the reliability of the disease modules obtained. The discovery of the same clusters using different methods indicates that these sub-modules are likely to be of biological relevance to the diseases characterized.

Although the complete landscape of heterogeneous disease is larger and more diverse than explored here, our results imply that locus heterogeneity genes show distinct properties allowing the identification of novel disease genes in the local network neighborhood, providing a pathway for further experimental study and candidate gene identification. Our finding that proteins encoded by locus heterogeneity disease genes are more highly interconnected than other types of disease genes indicates that clustering analysis will have particular value in identifying additional as yet unknown causative genes for diseases displaying locus heterogeneity. Increasing our understanding of specific gene classifications is essential to improve our knowledge of human disorders. As shown here, focusing on specific subsets of disease genes allows us to provide novel insights on a systems level to direct future research. As proteomic research continues, delivering a greater depth and reliability to human protein interaction data, we believe that studies such as this will become essential in providing novel advances to aid the identification of disease genes.

## Conflict of Interest Statement

The authors declare that the research was conducted in the absence of any commercial or financial relationships that could be construed as a potential conflict of interest.
